# BDH1 promotes lung cancer cell proliferation and metastases by PARP1‐mediated autophagy

**DOI:** 10.1111/jcmm.17700

**Published:** 2023-03-15

**Authors:** Zhimin Zhang, Xin Bi, Xiaojuan Lian, Zhongxi Niu

**Affiliations:** ^1^ Thoracic Surgery The Third Medical Center of PLA General Hospital Beijing China; ^2^ Department of Tumor Blood Chongqing University Jiangjin Hospital Chongqing China

**Keywords:** lung cancer, metastases, predictive marker, serum BDH1, therapeutic target

## Abstract

Lymph node metastases and distant metastases were the important limiting factor for therapy of unresectable locally advanced (IIIB stage) and oligotransduction (IVa stage) lung cancer. This study was undertaken to identify a novel predictive biomarker for predicting lymph node metastases of lung cancer. A total of 364 patients with lung cancer which comprised of 198 patients with transcriptome sequencing data, 110 cases with immunohistochemistry data and 66 patients with serum samples were included to identify and validate the candidate gene. Autophagy was measured by western blots, immunofluorescence and electron microscope. We found that 3‐hydroxybutyrate dehydrogenase 1 (BDH1) was associated with proliferation and metastases of lung cancer. BDH1 expression in both tissue and serum samples was associated with lung cancer metastases. Mechanical studies revealed that the AMPK‐mTOR signalling pathway mediated by PARP1 played an important role in BDH1‐induced autophagy. Activation of mTOR pathway markedly increased the effect of BDH1 in cell proliferation and metastases. These results were verified by the knockdown of PARP1. Furthermore, in vivo administration of BDH1 effectively promoted tumour growth in H460 xenografts mice. Our finding not only suggested that BDH1 might be useful as a novel biomarker and therapeutic target for lung cancer metastases, but also imply that PARP1‐mediated AMPK‐mTOR signalling pathway might play a critical role in BDH1‐induced autophagy and lung cancer proliferation and metastases.

## 1 INTRODUCTION

Lung cancer has become the leading cause of lethal and morbidity in the world. Radical resection is the best treatment of lung cancer. There are dramatic differences in prognosis between patients with surgical and non‐surgical treatments. About two thirds of lung cancer patients are newly diagnosed with unresectable locally advanced (IIIB stage) or oligotransduction (IVa stage), which were main limitation factors for radical resection of lung cancer. However, lymph node metastases and distant metastases were the important limiting factor for therapy of unresectable locally advanced (IIIB stage) and oligotransduction (IVa stage) lung cancer. Furthermore, the accurate diagnosis, especially the diagnosis of distant metastases, is one of the important factors to improve the prognosis of lung cancer. Only less than 80% accuracy in detecting lymph node metastases is achieved by enhanced CT, which is the first choice of diagnostic methods.^1^ Therefore, it is essential to look for a new biomarker for accurate diagnosis and targeted treatment.

In the study, we integrated transcriptome sequencing data of our clinical samples and an independent sample from TCGA data set of lung cancer with metastases to find 3‐hydroxybutyrate dehydrogenase 1 (BDH1) gene. The diagnostic value was further validated in tissue and serum samples. Moreover, high expression of BDH1 induced autophagy and subsequently proliferation and migration in lung cancer cells. PARP1‐mediated activation of AMPK‐mTOR signalling pathway plays an essential role in BDH1‐induced autophagy in lung cancer cells. Taken together, these findings suggested that BDH1 might be a useful novel biomarker and therapeutic target for lung cancer metastases, and that PARP1‐mediated AMPK‐mTOR signalling pathway played a critical role in BDH1‐induced autophagy, proliferation and metastases.

## 2 MATERIALS AND METHODS

### 2.1 Materials

Dulbecco's modified Eagle's medium (DMEM) and fetal bovine serum (FBS) were from Invitrogen (Carlsbad, CA, USA). Olaparib (PARP1 inhibitor AZD2281), rapamycin (Rapa), hydroxychloroquine (CQ), penicillin, streptomycin and dimethyl sulfoxide (DMSO) were from Sigma‐Aldrich (St Louis, MO, USA). Antibodies against BDH1, p62 and PARP1 were from Santa Cruz Biotechnology (Santa Cruz, CA, USA). Antibodies against BDH1, mTOR, LC3, LC3II, β‐tubulin and β‐actin were from Abcam (Cambridge, MA, USA). COMET Assay kit (R&D Systems; Trevigen), CCK‐8 assay, crystal violet, loading buffer and RIPA assay were from Beyotime Institute of Biotechnology (Haimen, China).

### 2.2 Cell culture

The NHI‐PC‐9 (PC‐9) and H460 cell lines were obtained from the American Type Culture Collection (ATCC; Manassas, VA, USA). The PC‐9 and H460 cell lines were cultured in containing 10% FBS, 50 mg/mL penicillin/streptomycin and 37°C humidified incubator with 5% CO_2_.

### 2.3 Transfection

The siRNA‐BDH1 (siBDH1) and siRAN‐con (siCON) plasmids and siRNA‐PARP1 (siPARP1) and siRAN‐con (siCON) plasmids were from Genechem (Shanghai, China). Using the Silencer siRNA construction kit (Qiagen, Venlo, The Netherlands), the cells were incubated with siRNA transfection complexes, and were performed with the RNAiFect reagents (Qiagen, Venlo, The Netherlands). The cells were used for the experiments 48 h after transfection (Hagan, Yacoub, & Dent, 2007).

### 2.4 Western blot

The cells were collected and the protein was extracted using the RIPA buffer (Beyotime Biotechnology, China). Protein concentration was determined using the BSA assay. Equal amounts of proteins were separated on SDS‐polyacrylamide gels and transferred to PVDF membranes. The membranes were blocked with 5% non‐fat dry milk in TBST for 1 h at room temperature. The membranes were incubated with the primary antibody against PARP1, mTOR, AMPK, LC3, P62, BDH1, p‐AMPK (ab267373, 1:1000, Abcam), p‐mTOR and β‐tubulin and then with HRP‐labelled anti‐rabbit IgG secondary antibodies (No. 7074, 1:5000; CST, Danvers, MA) overnight at 4°C, followed by 1 h at room temperature with HRP‐conjugated secondary antibodies. Protein bands were visualized with BioMax‐Light films (Eastman Kodak Co., Rochester, NY, USA). The protein bands were analysed using a Gel Doc 2000 apparatus and the Quantity One software (Bio‐Rad, Hercules, CA, USA).

### 2.5 Cell proliferation, scratch assay and transwell assay

Cells were seeded in 6‐well plates at a density 2 × 10^4^ /well. At 24 h following growth, the cells were treated as indicated. Cell viability was detected using microscope and collection of pictures. Cells reach a density of 70%. Cell monolayers were then scratched with a 100 μL yellow pipette tip and washed with PBS three times to remove detached cells. The wounded areas were imaged using an Olympus microscope and marked. The indicated doses of palbociclib, celecoxib or LPS were applied to culture cells for 48 h at 37°C in 5% CO_2_. The same areas were imaged again to observe the wound gap. The Matrigel invasion assay was carried out using 8 μm pore size transwell filters (Corning, 6.5 mm diameter). The cell culture inserts were coated with 5 μL pure Matrigel (Sigma, St. Louis, MO) and placed in a 24‐well plate. Celecoxib was added at constant concentration to both the upper and the lower chambers. After 18 h of incubation in 5% CO_2_ at 37°C, the filters were fixed with 90% ethanol for 30 min and stained with crystal violet. The invasive cells on the lower surface of the filters were examined by bright field microscopy.

### 2.6 Clonogenic assay

A clonogenic assay was used to determine the survival and proliferation of the different cell lines after drug treatment.^2^ Cells were plated on 60 mm plastic dishes 1 day before drug treatment in order to produce approximately 500 surviving colonies. And each group was inoculated with three wells and cultured in an incubator for 13 days. The formation of clones was observed under a low‐power microscope, and the culture was terminated if the number of cells per colony was >50. The supernatant was discarded, and the cells were gently washed in room temperature with PBS twice. Then, 4% paraformaldehyde was added to fix the cells at room temperature for 30 min. The fixative was removed, and then 0.2% crystal violet was added for staining at room temperature in the dark for 30 min. The crystal violet was discarded, the cells were gently washed with PBS once or twice and the cells were naturally air‐dried. For colony counting, the air‐dried colony formation plate was placed on white paper painted with a grid, and the colonies were counted with the naked eye. The experiment was repeated three times.

### 2.7 Electron microscopy

Cells were washed and centrifuged; then, the cells were stained with 2% uranyl acetate in water in the dark. Sequential ethanol series were used to dehydrate the samples. Sections were made (70 nm) and stained with lead citrate and uranyl citrate. Zeiss microscope performed transmission electron microscopy analysis (Carl Zeiss GmbH, Oberkochen, Germany).

### 2.8 In vivo experiment

Female BALB/C nude mice (6–8 weeks of age) were obtained from the Daping Hospital of Army Medical University Laboratory Animal. The mice were housed in a pathogen‐free environment under controlled conditions (temperature: 20–26°C, humidity: 40%–70%, light/dark cycle 12/12 h). The mice were injected subcutaneously with 3 × 10^6^ H460 cells. Tumour diameter was measured twice a week using callipers. Tumour size was calculated as ab^2^/2 mm^3^ (where a is the length and b is the width of the tumour). Mice with tumours reaching about 100 mm^3^ were randomized into two groups (10/group): siBDH1 group and siCON group.^3^ The protocol was approved by the Ethics Committee of the Daping Hospital and Research Institute, Army Medical University.

### 2.9 TCGA data set

A preprocessed expression matrix of gene‐level RSEM values documented in TCGA data sets (Table. S2 Clinical characteristics of lung cancer patients) (https://tcga.xenahubs.net/download/TCGA.LUNG.sampleMap/AgilentG4502A_07_3.gz). Clinical information of this TCGA cohort were obtained from UCSC (https://tcga.xenahubs.net/download/TCGA.LUNG.sampleMap/LUNG_clinicalMatrix.g).

### 2.10 RNA preparation, qualification and transcriptome sequencing

Total RNA was isolated with RNeasy plus Mini Kit (Catalogue no. 74134, Qiagen, Duesseldorf, German). RNA concentration and integrity were detected by the RNA Nano 6000 Assay Kit of the Agilent Bioanalyzer 2100 system (Agilent Technologies). A total amount of 5 μg RNA per sample was used as input material for the RNA sample preparations. Sequencing libraries were generated using NEBNext®Ultra™ RNA Library Prep Kit for Illumina® (NEB, USA) following manufacturer's recommendations and index codes were added to attribute sequences to each sample. The clustering of the index‐coded samples was performed on a cBot Cluster Generation System using TruSeq PE Cluster Kit v4‐cBot‐HS (Illumia) according to the manufacturer's instructions. After cluster generation, the library preparations were sequenced on an Illumina Hiseq 2500 platform and paired‐end reads were generated. Index of the reference genome was built using Bowtie v2.2.3 and the reads were blasted to the reference genome using TopHat v2.0.12. The expression level of each gene was conducted based on RNA‐seq and was measured as numbers of reads per kilobase of exon region in a gene per million mapped reads (RPKM). Differential expression analysis of two samples was performed using the DEGseq (2010) *R* package. *p* value was adjusted using *q* value [19]. FDR <0.05 and |log2Ratio| ≥1 found by DESeq was set as the threshold for significantly differential expression.

### 2.11 Real‐time PCR

The expression levels of the 58 genes were validated by real‐time RT‐PCR. The cDNA was synthesized with RevertAid Fist Strand cDNA Synthesis Kit (ThermoFisher, United States, Category Number: #K1622). Primers purchased from Bioligo Inc. (Shanghai, China). Real‐time quantitative RT‐PCR was performed with the SYBR Green PCR method using Fast SYBRTM Green Master Mix (ThermoFisher, United States, Category Number: 4385610) with CFX96TM Real‐Time System (Bio‐Rad, United States). GAPDH was used as endogenous normalization controls. 2^−△△Ct^ method was used to calculate the expression levels. Real‐time PCR was performed in duplicate in three dependent experiments.

### 2.12 ELISA analysis

Indirect ELISA was used to detect serum BDH1 and carried out as follows: Microtiter plates (450, 500 ng/cm^2^, 8‐well 612 strips, Costar Biosciences Inc, USA) were coated with rabbit antibody of BDH1 with coating buffer (0.1 mol/L carbonate buffer, pH 9.6) at 1.0 mg/mL and incubated at 4°C overnight; Plates were washed three times using washing buffer (0.05% PBST), mouse antibody of BDH1 with coating buffer (0.1 mol/L carbonate buffer, pH 9.6) at 1.0 mg/mL and incubated for 2 h at room temperature; After three washes, 100 mL/well of serum samples were incubated for 2 h at 37°C, PBS and a standard product was used as the calibration control; The unbound compounds were washed away; A total quantity of 50 mL/well of HRP‐labelled goat anti‐human IgG (working concentration recommend 1:5000 dilution) were incubated for 30 min at 37°C; After washing three times, 50 mL/well of TMB (Pierce, USA) substrate solution was incubated for 10 min at room temperature; the enzymatic reaction was stopped by 2 mol/L H_2_SO_4_ (50 mL/well) and then optical density (OD) was measured by microplate spectrophotometry at reference wavelength (450 nm). All samples were tested twice in two separate plates.

### 2.13 IHC analysis

The expression of BDH1 and β‐catenin proteins in human specimens was measured by IHC. After deparaffinization and blocking, slides were incubated with BDH1 and P62 monoclonal antibodies (1:100 and 1:50 dilution) overnight at 4°C and then incubated with a 1:50 dilution of goat anti‐mouse secondary antibody for 1 h at room temperature. Finally, slides were incubated with 3,30‐diaminobenzidine (DAB) substrate. Scoring for BDH1 and P62 staining was performed as described previously by three professional pathologists.

### 2.14 Patients, tissue and serum specimens

Ten specimens used to transcriptome sequencing and bioinformatics analysis were obtained from surgically resected primary tissue in patients with lung cancer treated at Daping Hospital of the Army Medical University (China) and were frozen and kept in liquid nitrogen (Table. S4 Clinical characteristics of lung cancer patients). The tumour blocks which were subjected to immunohistological analysis was comprised of two cohorts, one was 80 patients treated at Daping Hospital of the Army Medical University (China) and another one was 30 patients treated at Wuhan Union Hospital of Huazhong University of Science and Technology (China) (Table. S5 Clinical characteristics of lung cancer patients). Tissue samples were collected after the first‐line radical resection of primary tissue of lung cancer. The serum samples which were subjected to ELISA analysis: 66 patients treated at Daping Hospital of the Army Medical University (China) (Table. S6 Clinical characteristics of lung cancer patients). Serum samples were collected before any antitumour therapy of lung cancer. The Ethics Committee of the Daping Hospital and Research Institute, Army Medical University approved the study protocol.

### 2.15 Statistical analysis

Fifty‐eight significantly differentially expressed genes were analysed to identify candidates potentially predictive for lymph node metastases using univariate logistic regression. Nine genes including DDX49, USH1G, MRM1, RBM28, USP49, BDH1, EGFR, ABHD11 and GTPBP3 were found associated with lymph node metastases with crude *p* ˂ 0.05. Independent predictors for lymph node metastases were analysed by stepwise logistic regression analysis with age, gender, histology, T and clinical stage and nine candidate genes as initial covariates (Table. S3). The variables were selected by likelihood ratio test according to the following criteria: a variable with a *p* value lower than 0.05 was selected, whereas a variable with a *p* value higher than 0.1 was excluded during backward deletion steps. The expression of EGFR, BDH1 and stage was independent predictors for lymph node metastases. The calculated predictive probability in multivariate logistic regression was used as the risk score for lymph node metastases. ROC analysis was used to evaluate the discriminative efficiency of the expression of individual gene and risk score for lymph node metastases. The area under the ROC curve was compared using function ‘*roc.test*’ embedded in R language package ‘*pROC*’ (version 3.4.4 Foundation for Statistical Computing, Vienna, Austria) with method proposed by DeLong (DeLong, DeLong, & Clarke‐Pearson 1988), and Holm–Sidak procedure was used for multiple comparison correction. All other statistical analyses were performed using SPSS 17.0 (IBM SPSS, Chicago, IL, USA). All tests were bilateral, and *p* < 0.05 was considered statistically significant.

## 3 RESULTS

### 3.1 BDH1 was a biomarker of lung cancer metastases in tissue and serum samples

**FIGURE 1:**
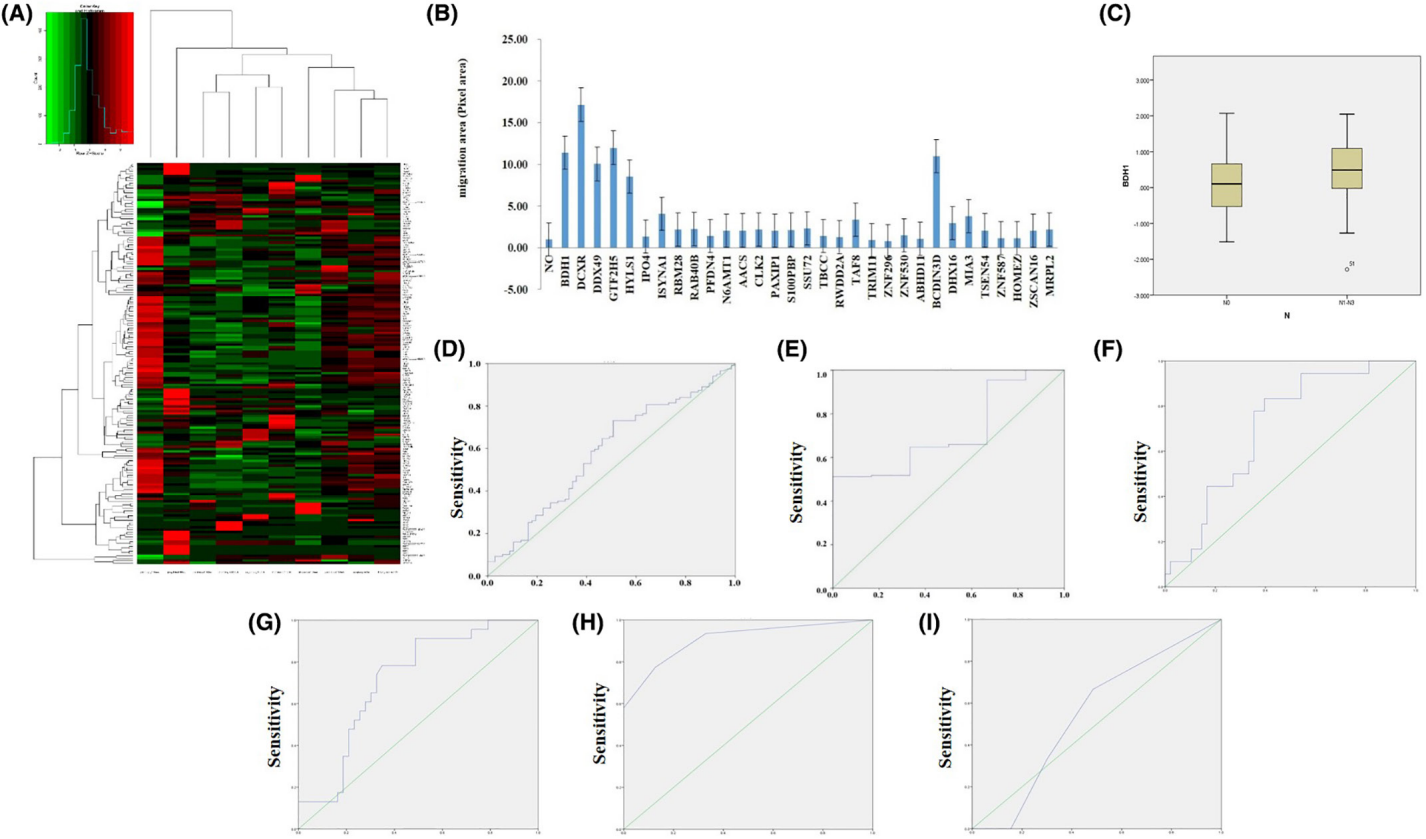
BDH1 was a biomarker of lung cancer metastases in tissue and serum samples. (A) The 169 genes differentially expressed in five paired positive and negative lymph node metastases from transcriptome sequencing; (B) nine genes were associated with cell migration in lung cancer PC‐9 cells, data represent the knockdown experiment of 58 genes. (C) BDH1 expression was higher in primary lung cancer tissue from patients with lymph node metastases (N1‐N3) than in those without (N0). (D) The ROCs of the BDH1 obtained using logistic regression with risk factors. The AUC, sensitivity and specificity of BDH1 for prediction of lymph node metastases were 63.6%, 65.2% and 63.9% respectively. (E) The AUC, sensitivity and specificity of BDH1 for prediction of distant metastases were 71.5%, 64.8% and 66.7% respectively. (F) The AUC in the 110 tissue samples for the prediction of lymph node metastases was 90.8%. (G) The AUC in the 110 tissue samples for the prediction of distant metastases was 54.5%. (H) The AUC in the 66 serum samples for the prediction of lymph node metastases was 70.9%. (I) The AUC in the 66 serum samples for the prediction of distant metastases was 71.4%.

To research the molecular mechanism underlying metastases of lung cancer, a sequential approach was used to identify genes associated with lymph node metastases. A total of 169 genes differentially expressed in positive versus negative lymph node metastases were firstly identified (Figure 1A); Among them, 58 genes were expressed in lung cancer PC‐9 cell line (Figure. S1); nine genes (BDH1, DCXR, DDX49, GTF2H5, HYLS1, ISYNA1, TAF8, BCDIN3D and MIA3) were associated with cell migration in lung cancer cell lines (Figure 1B),^4^ These nine genes were further analysed in TCGA data set of 188 samples with transcriptome sequencing results (Figure 1C,D,E). Three genes (BDH1, DDX49 and EGFR) were associated with lymph node metastases of lung cancer, and BDH1 gene had a highest predictive capacity for lymph node metastases and distant metastases through multivariate logistic regression.

To identify the predictive value of BDH1 for lymph node metastases and distant metastases of lung cancer, we analysed the exon sequencing data of 188 patients with lung cancer from TCGA and obtained three genes that were associated with lymph node metastases and cell migration, with a crude *p* ˂ 0.05, through multivariate logistic regression. Transcriptome sequencing results verified that the mRNA expression values of BDH1 in patients with lymph node metastases were significantly higher than those in patients without lymph node metastases with a median (IQR) of 0.490 (−0.025–1.093) versus 0.102 (−0.540–0.663) (*p* = 0.002) respectively (Figure 1D). The prediction rate (area under the curve [AUC]), sensitivity (true positives) and specificity (false positives) of BDH1 mRNA expression level for the prediction of lymph node metastases and distant metastases were 63.6%, 65.2%, 63.9% and 71.5%, 64.8%, 66.7% respectively (Figure 1D,E). These results suggested that BDH1 gene is significantly correlated with lymph node metastases of lung cancer.

To validate the predictive value of BDH1 expression for lung cancer metastases, we analysed the association of the protein expression with the risk of lymph node metastases in two independent cohorts of a total number of 176 lung cancer patients with tissue immunohistochemistry (IHC) and serum ELISA assay. The clinical characteristics of these 176 patients were shown in Tables S1 and S2. Firstly, we analysed 110 tumour specimens by IHC obtained from patients who had been diagnosed by enhanced CT and/or PET–CT and pathology. Univariate logistic regression analysis demonstrated that patients with a high expression of BDH1 (++/+++) had significantly higher lymph node metastases rate than those with low expression (−/+) (++/+++ vs. −/+: OR = 23.36, 95% CI: 8.10–69.09, *p* < 0.001). The AUC for the prediction of lymph node metastases and distant metastases were 70.9% and 71.4% respectively in this cohort (Figure 1F,G).

Serum analysis is a less invasive approach for tumour diagnosis. We found that BDH1 was expressed in both fresh serum and frozen serum by dot blot analyses. We collected 66 serum samples from lung cancer patients before any anticancer therapy whose clinical characteristics were shown in Table S2. The indirect ELISA method was set up and used to detect concentration of serum BDH1 protein. Among the 66 lung cancer patients, the serum concentration of BDH1 was significantly higher in patients with metastases than that in those without any metastases (median [IQR]: 311.5 [243.3–388.4] ng/mL vs. 104.7 [164.3–205.8] ng/mL, *p* < 0.001). The higher concentration of BDH1 in serum was strongly associated with a higher risk of node metastases (OR = 1.462 per 20 ng/mL increase, *p* = 3.63 × 10–4). The discriminative capacity of serum BDH1 for lymph node metastases and distant metastases evaluated with ROC analysis had 90.8% and 54.5% AUC respectively (Figure 1H,I). These results suggested that serum BDH1 expression was a meaningful potential diagnostic biomarker of lymph node metastases. Taken together, these results demonstrated that the tissue and serum expression levels of the candidate protein had a high predictive capacity for the detection of lung cancer metastases in our clinical samples.

### 3.2 Role of BDH1‐mediated autophagy in cell proliferation and metastases

**FIGURE 2:**
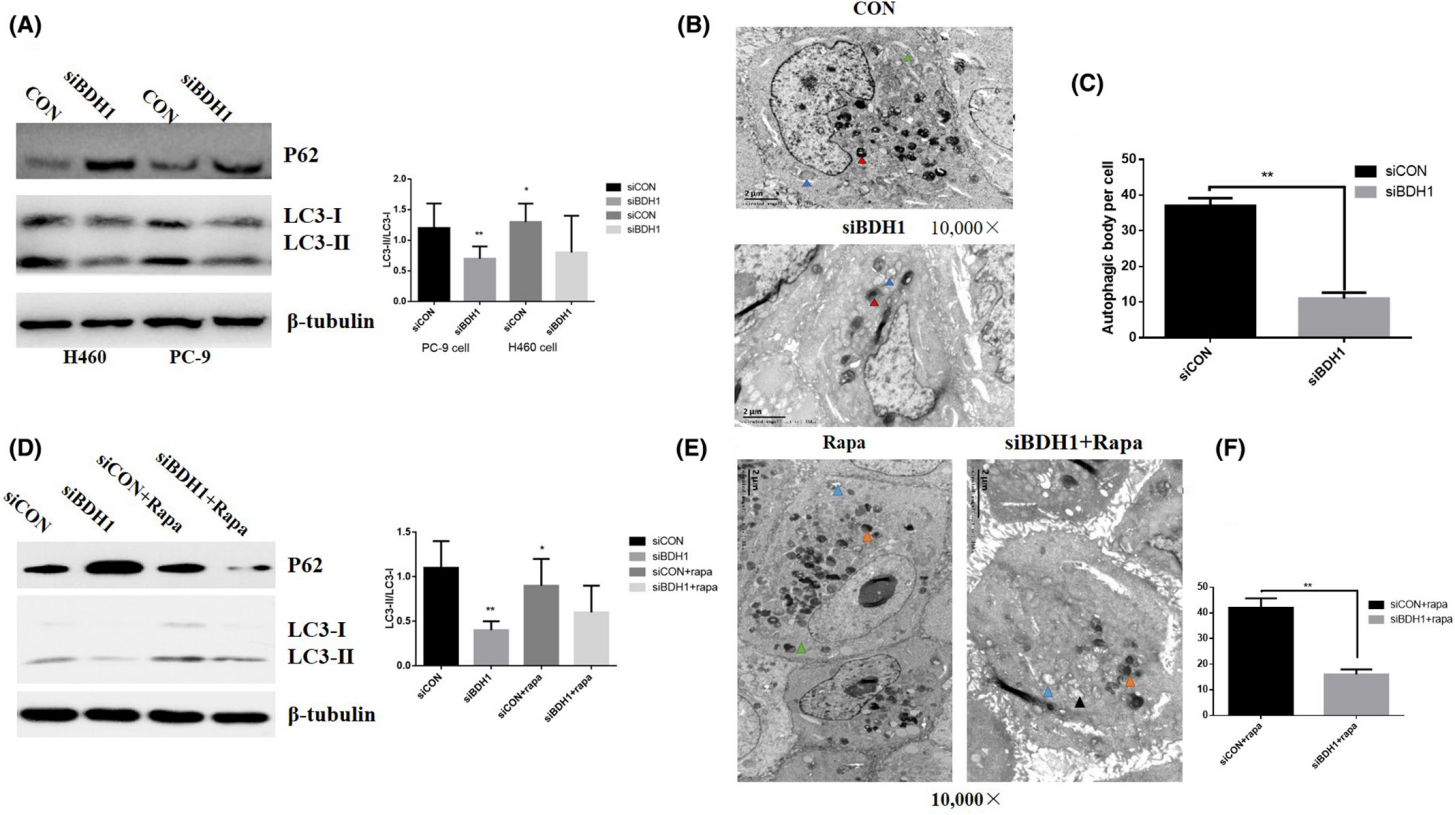
Role of BDH1‐mediated autophagy in cell proliferation and metastases. (A) Knockdown of BDH1 by siRNA‐BDH1 (siBDH1) in PC‐9 and H460 cells. Western blot measured protein expression. (B, C) Electron microscope assay determined autophagy level in PC‐9 cells. (D) Rapamycin increased the ratio of LC3‐II/LC3‐I in PC‐9 cells. Western blot measured protein expression. (E, F) Electron microscope assay determined autophagy level in PC‐9 cells. Red triangle: autophagosome; blue triangle: lysosome; black triangle: Golgi apparatus; green triangle: endoplasmic reticulum. Data represent the average of three independent experiments (mean ± SD). **p < 0.001.

Our results suggested that BDH1 had direct role on cell growth, migration and invasion. More importantly, we found that the gene had autophagy‐induced activity. As shown in Figure 2A, downregulation of BDH1 significantly reduced the expression of LC3‐II and significantly increased in the expression level of P62 by a lentivirus‐knockdown assay in starved PC‐9 cells and H460 cells for 4 h. Then, the role of BDH1 in autophagy was further confirmed by immunofluorescence (Figure. S2) and electron microscopy (Figure 2B,C). BDH1‐induced autophagy might play an important role in cell proliferation and migration.

In order to uncover the relationship between BDH1‐induced autophagy and lung cancer progression, PC‐9 cells were treated with 10 mM rapamycin and siBDH1. The expression of LC3‐II markedly reduced, whereas the P62 expression was found to be increased. We then treated the PC‐9 cells with rapamycin and siCON or siBDH1 (Figure 2D). The results from immunofluorescence (Figure. S2) and electron microscopy (Figure 2E,F) showed that the level of LC3‐II and autophagic body were higher in siBDH1 + rapamycin group compared with non‐rapamycin group and rapamycin significantly enhanced BDH1‐induced autophagy. These results indicated that rapamycin‐induced autophagy might be the downstream signalling of BDH1.

### 3.3 Effects of rapamycin on BDH1‐mediated lung cancer cell growth and migration

**FIGURE 3:**
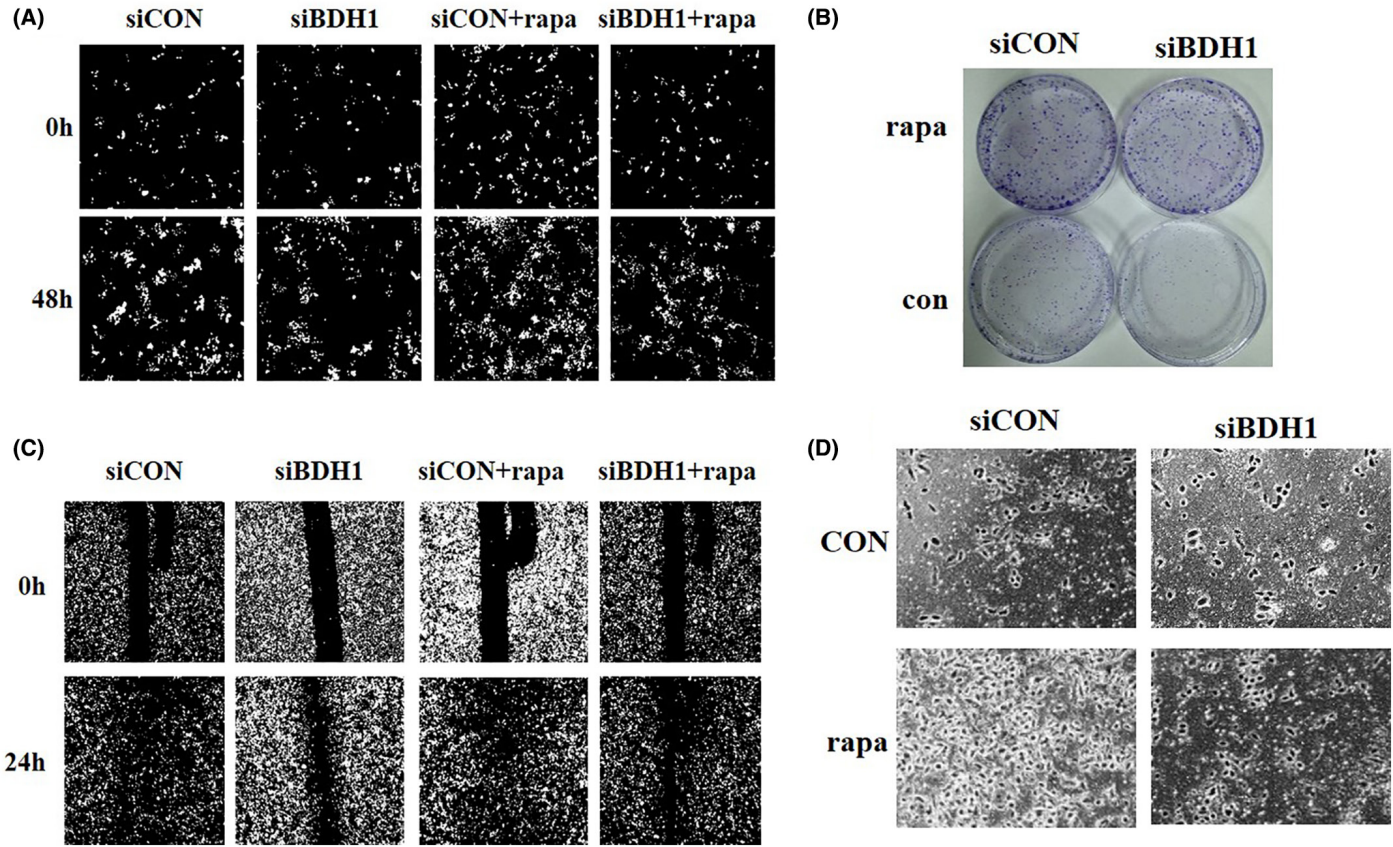
Rapamycin (Rapa) promoted cell growth, migration and invasion after siRNA‐BDH1 in vitro. (A) Rapamycin treatment in H460 cells significantly increased cell proliferation (A and B), migration (C) and invasion (D) abilities induced by downexpression of BDH1, compared with that of control cells. Rapamycin treatment in H460 cells significantly increased cell proliferation (A, B), (C) migration and invasion (D) abilities induced by downexpression of BDH1, compared with that of control cells. Data represent the average of three independent experiments (mean ± SD).

To explore the role of BDH1‐induced autophagy in proliferation and metastases in lung cancer cells, we treated PC‐9 cells with rapamycin and lentivirus transfection. Our results showed that rapamycin treatment substantially increased cell growth (Figure 3A,B), migration and invasion (Figure 3C,D). Rapamycin markedly increased the capacity of cell growth, migration and invasion in BDH1 downregulation cells. These results indicated that rapamycin treatment reversed the inhibition of proliferation and migration induced by BDH1 downregulation.

### 3.4 BDH1 induced autophagy though activation of PARP1‐mediated AMPK‐mTOR signalling pathway

**FIGURE 4:**
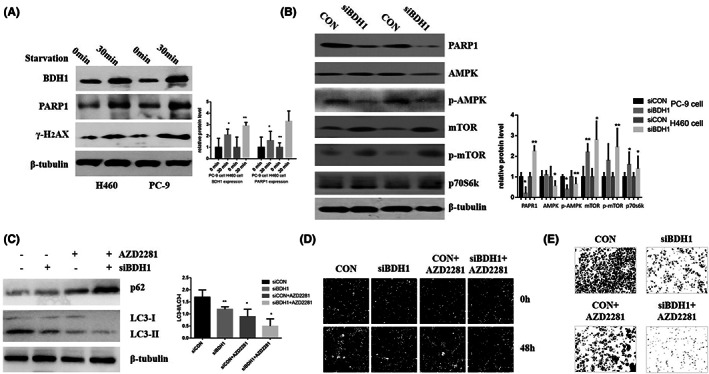
Effects of BDH1 on lung cancer cell PARP/AMPK/mTOR/autophagy signalling pathway in PC‐9 cells. (A) Western blot showed that starvation increased downregulation of BDH1, decreased the levels of BDH1 and PARP1 signalling pathway in PC‐9 cells and H460 cells. (B) Western blot showed that downregulation of BDH1 decreased the levels of PARP1 and p‐AMPK/mTOR signalling pathway in PC‐9 cells. (C) Western blot showed that PARP1 inhibitor (AZD2281) enhanced the effect of siRAN‐BDH1 transfection on decreased ratio of LC3‐II/LC3‐I and the increased P62 expression in PC‐9 cells. AZD2281 treatment in PC‐9 cells significantly enhanced proliferation inhibition (D) and (E) migration abilities induced by downexpression of BDH1, compared with that of control cells. Data represent the average of three independent experiments (mean ± SD).

KEGG pathway enrichment analysis of all differentially expressed genes showed that the PI3K/Akt pathways might play crucial roles in the metastases of lung cancer (Figure. S3, Table S3). Cell starvation induced a decrease in the expression level of BDH1 and PARP1 (Figure 4A). We demonstrated that BDH1 downregulation decreased PARP1, AMPK and mTOR activation (Figure 4B). These findings indicated that decrease in the activation of AMPK/mTOR signalling by BDH1 knockdown might play a key role in inhibiting cell proliferation and migration in vitro and PARP1 inhibitor decreased the LC3‐II expression (Figure 4C), cell proliferation and invasion (Figure 4 D,E) and increased P62 expression (Figure 4C) induced by BDH1 downregulation in PC‐9 cells. These results suggested that the activation of PARP1/AMPK/mTOR pathway might play an important role in autophagy, proliferation and metastases in cooperation with BDH1 expression.

### 3.5 Effects of PARP1/AMPK/mTOR/autophagy pathway on cell growth and migration

**FIGURE 5:**
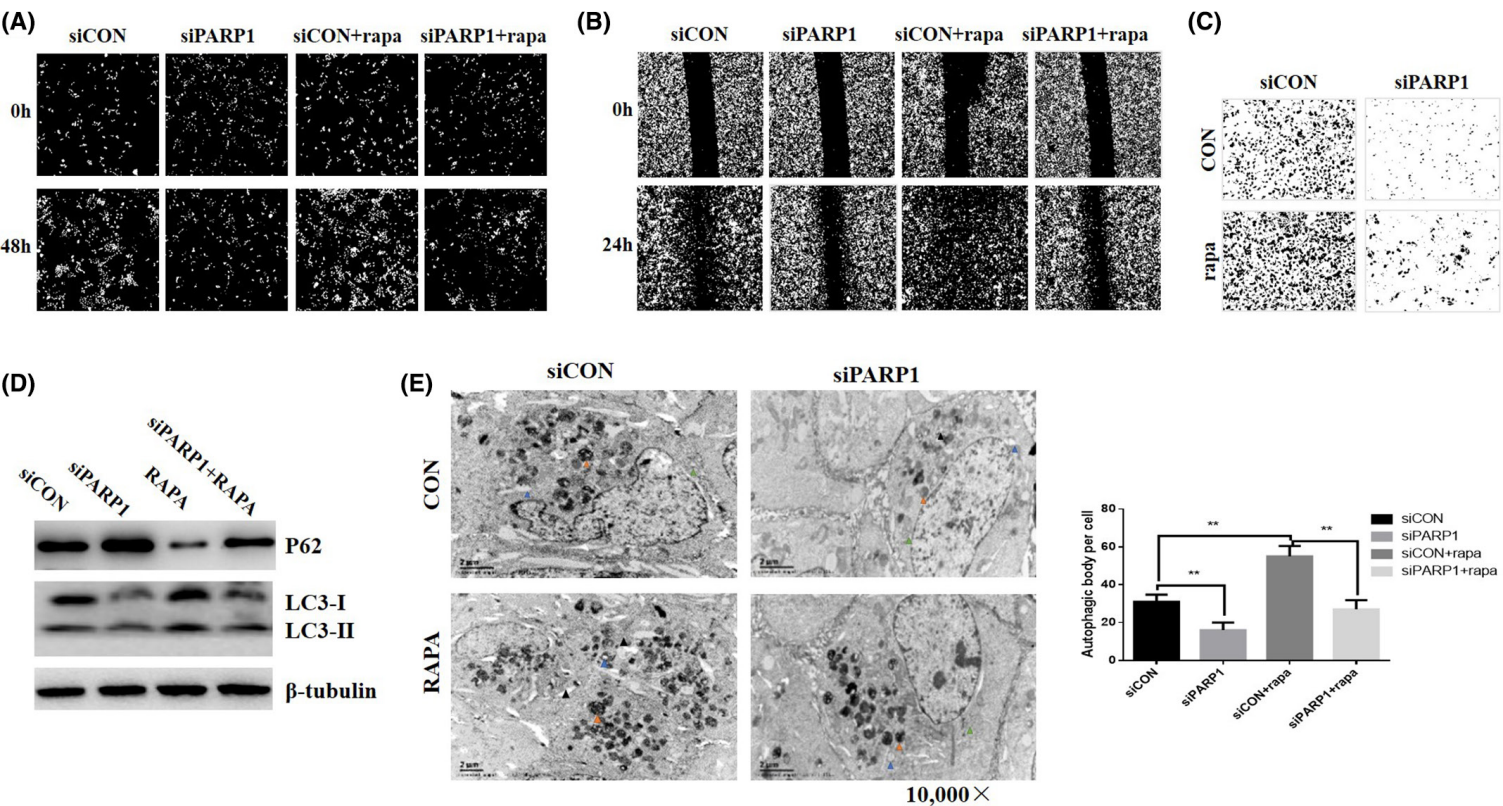
Effects of PARP1/mTOR/autophagy signalling pathway on lung cancer cell growth, migration and invasion. Rapamycin (Rapa) treatment in PC‐9 cells significantly reversed the inhibition of cell proliferation (A), migration (B) and invasion (C) abilities induced by siRNA‐PARP1 transfection, compared with that of control cells. Rapamycin (Rapa) treatment in PC‐9 cells significantly reversed the inhibition of cell autophagy, as shown (D), Rapamycin treatment increased ratio of LC3‐II/LC3‐I, but decreased P62 expression. SiRNA‐PARP1 transfection decreased ratio of LC3‐II/LC3‐I, but increased P62 expression. (E, F) Electron microscope assay determined autophagy level in PC‐9 cells. Red triangle: autophagosome; blue triangle: lysosome; black triangle: Golgi apparatus; green triangle: endoplasmic reticulum. Data represent the average of three independent experiments (mean ± SD). **p < 0.001.

To explore the role of PARP1/AMPK/mTOR pathway in cell proliferation and metastases in lung cancer, we treated PC‐9 cells with rapamycin and lentivirus transfection. PARP1 knockdown substantially decreased cell growth, migration and invasion (Figure 5 A‐C). In contrary, rapamycin reversed the effect induced by PARP1 knockdown. Rapamycin markedly increased the capacity of cell growth, migration and invasion in PARP1 downregulation cells. These results indicated that rapamycin treatment reversed the inhibition of proliferation and migration induced by PARP1 downregulation.

As shown in Figure 5D, these cells were starved with Hank buffer for 4 h, the downregulation of PARP1 significantly reduced the expression level of LC3‐II in PC‐9 cells using a lentivirus‐knockdown assay and significantly increased in the expression level of P62. However, treatment with rapamycin reversed the effect of PARP1 knockdown. The role of PARP1 in autophagy was further verified by immunofluorescence and electron microscopy (Figure. S4E,F). PARP1‐induced autophagy was enhanced by rapamycin treatment; PARP1/AMPK/mTOR pathway might play an important role in lung cell autophagy.

### 3.6 Downregulation of BDH1 inhibited tumour growth in vivo

**FIGURE 6:**
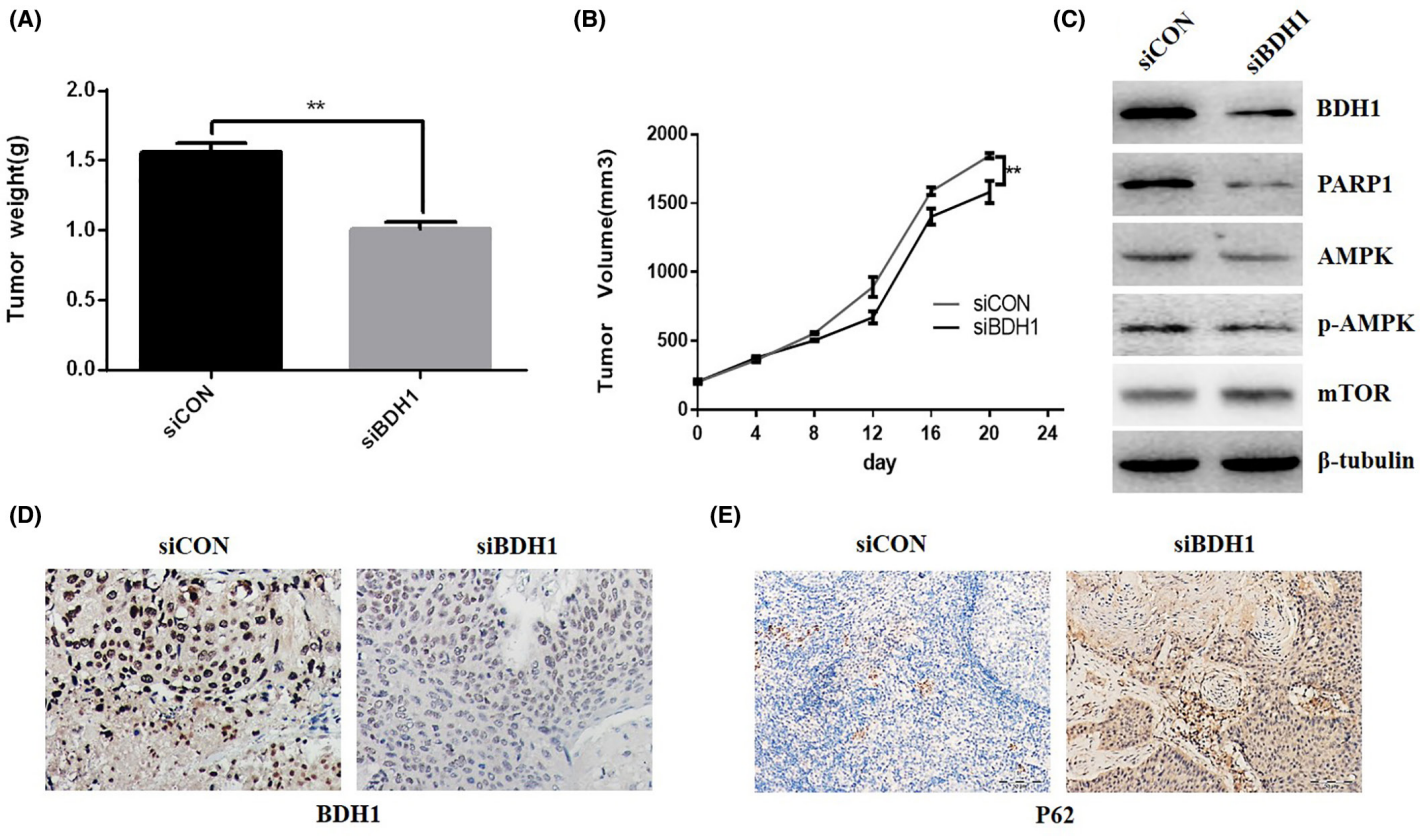
Effects of BDH1 on lung cancer cell growth in vivo. Nude mice were injected with H460 cells stably transfected with a plasmid containing shBDH1. Xenograft models were divided into two groups (10 mice/group), and then the nude mice were fed for 14 days. Tumour weight was measured on day 14 (A). Tumour volume was measured using a calliper (B). Tumour tissues isolated from xenografts were subjected to western blot analysis (C) and immunohistochemistry analysis (D, E). The data are shown as the mean ± standard error, *n* = 10. **p < 0.01 versus the vehicle group.

We studied the effect of BDH1 expression on cell growth by injecting H460 cells which were transfected with control (siCON) or BDH1‐downregulation vector (siBDH1) into nude mice in vivo. We observed that downregulation of BDH1 significantly decreased tumour growth in vivo (Figure 6A,B). Consistent with observations in vitro, transfection with siBDH1 significantly decreased BDH1 expression in vivo (Figure 6D) and the activation of PARP1/AMPK/mTOR signalling pathway (Figure 6C) as well as P62 expression (Figure 6E). These findings indicated that downregulation of BDH1 decreased the activation of PARP1‐AMPK‐mTOR‐autophagy signalling and might play a key role in inhibiting cell proliferation and migration in vivo.

## 4 DISCUSSION

Current treatments for lymph node metastases and distant dissemination merely have low local remission rate and poor efficacy, accurate diagnosis and effective treatment strategy need to be explored. In this study, we integrated transcriptome sequencing data of our clinical samples and an independent sample from TCGA data with lung cancer metastases to find a novel candidate biomarker and validated the clinical value in tissue and serum samples. Moreover, BDH1 can induce autophagy and subsequent proliferation and migration in lung cancer cells. PARP1‐mediated activation of AMPK‐mTOR signalling pathway plays an essential role in BDH1‐induced autophagy in lung cancer cells. Taken together, these findings suggested that BDH1 might be a useful novel biomarker and therapeutic target for lung cancer metastases, and that PARP1‐mediated AMPK‐mTOR signalling pathway might play a critical role in BDH1‐induced autophagy, proliferation and metastases.

The selection of BDH1 gene in the microarray detection cohort and other independent cohort of 188 patients from TCGA data set, and the patterns of gene expression found on microarray analysis were also validated by RT‐PCR. In addition, the discriminative capacities of BDH1 gene were validated by ROC analysis in three independent cohorts from patients with lung cancer. The direct link between cell migration and BDH1 expression was observed by gene knockdown assay. Thus, we believed that the BDH1 as potential biomarker for predicting lymph node metastases in lung cancer patients is reliable.

Our data suggested that the serum BDH1 was an important biomarker for metastases of lung cancer. We identified BDH1 gene through a series of strict process, including four independent cohorts, functional verification in two kinds of lung cancer cell lines and a series of cell function experiments. Firstly, the mRNA expression of BDH1 was associated with lymph node metastases of lung cancer by our transcriptome sequencing data and TCGA data set. There was a significant correlation between mRNA of BDH1 expression and distant metastases of lung cancer. Furthermore, BDH1 expression in both tissue and serum was an important predictive biomarker of lymph node and distant metastases of lung cancer. Secondly, knockdown of BDH1 decreased proliferation, migration and invasion in lung cancer cell lines. Although no rigorous validation set was included into the analysis, a series of functional verification in cell lines and reproducible results from the tissue and serum samples could demonstrate that BDH1 was an important biomarker of metastases of lung cancer.

BDH1 was upregulated in cancer and could serve as a critical therapeutic target for metastases of lung cancer. BDH1 was ketolytic key enzymes. In the mitochondrion, BDH1 catalyses βHB converting to AcAc and then AcAc is transformed into AcAc‐CoA in a near‐equilibrium catalysed reaction. Finally, AcAc was converted into AcAc‐CoA.^4^ Our results found that BDH1 was an important gene for lymph node metastases and distant metastases of lung cancer, and BDH1 knockdown reduced cell proliferation, migration and invasion. Punit Saraon proposed that enzymes of the ketogenic pathway including BDH1 might be important biomarkers in tissue for the diagnosis or prognosis of high‐grade cancer.^5^ Martinez‐Outschoorn, U. E. reported that BDH1 was critical for promoting tumour growth and metastases by ketolysis.^6^ Those results indicated that BDH1 expression promoted metabolism of ketone bodies to provide energy for cancer cell and activate the signalling pathway of cell proliferation, migration and invasion.

BDH1 might be not only involved in the metabolites of ketone bodies, but also have important cellular signalling roles. It was unknown whether BDH1 promoted the proliferation, migration and invasion of lung cancer cells through some signalling pathways independent of its role in ketone body metabolism. Martinez‐Outschoorn, U. E. report that fibroblasts overexpressing BDH1 show increased autophagy which was verified by two kinds of methods.^6^ Ketone body utilization drives tumour growth and metastases.^6^ BDH1 could serve as a target for ketone inhibition to effectively treat advanced cancer patients with tumour recurrence and metastatic disease.^6^ However, our results shown that BDH1 downregulation reduced the proliferation, migration, invasion and autophagy by PARP1‐mediated AMPK‐mTOR signalling pathway. In addition, we found that PARP1 inhibition decreased the proliferation, migration, invasion and autophagy. PARP1‐mediated cytoprotective autophagy during the response to DNA damage had been confirmed.^7^, ^8^ Ketone body metabolism can potentially enhance PARP1‐mediated NAD(+)‐related mechanisms.^9^ Although there was no direct evidence of regulatory mechanism between BDH1‐mediated ketone body metabolism and PAPR1 signalling pathway, we believed that ketone body metabolism caused by BDH1, at least BDH1 expression, promoted cell proliferation, migration, invasion and autophagy via PARP1‐mediated AMPK‐mTOR signalling pathway.

The PARP1/AMPK‐mTOR signalling pathway plays an important role in the proliferation and metastases in lung cancer cell. The PARP1/AMPK‐mTOR signalling pathway was a key regulator of the initial steps of autophagy, which protect cell from reactive oxygen species stress.^8^ Autophagy via AMPK activation downstream of PARP‐1 activation blocked cell death and played pro‐survival function of autophagy in cells.^10^ Yoon, J. H. reported that autophagy through DNA repair regulated by AMPK activation of PARP‐1 contributed to the sustained survival of breast cancer cells.^11^ Our results also confirmed that PARP1/AMPK‐mTOR‐mediated autophagy played pro‐survival function and promoted the migration and invasion in lung cancer cell. These results suggested that autophagy mediated by PARP1/AMPK‐mTOR signalling pathway promoted cell proliferation and metastases, which might be related to tumour energy metabolism in lung cancer.

Taken together, serum BDH1 was a useful biomarker for the lymph node metastases and distant metastases of lung cancer. Downregulation BDH1 significantly reduced the proliferation, migration and invasion. BDH1 might be a novel therapeutic target for advanced stage lung cancer. In addition, BDH1, through activating PARP1‐mediated AMPK‐mTOR signalling pathway promoted lung cancer progression. In the future, BDH1 might be a useful biomarker for the diagnosis and therapy of lung cancer.

## AUTHOR CONTRIBUTIONS

**Zhimin Zhang:** Conceptualization (equal); data curation (equal). **Xin Bi:** Software (equal); supervision (equal). **Xiaojuan Lian:** Data curation (equal); resources; validation (equal). **Zhongxi Niu:** Resources; supervision.

## FUNDING INFORMATION

The National Nature Science Foundation of Chongqing (cstc2021jcyj‐msxmX1223) supported this work.

## CONFLICT OF INTEREST STATEMENT

No potential conflicts of interest were disclosed.

## CONSENT FOR PUBLICATION

Written informed consent for publication was obtained from all participants.

## Supporting information


Appendix S1.

## Data Availability

Data and materials will be shared.
