# Supplementary material for: BDH1 promotes lung cancer cell proliferation and metastases by PARP1‐mediated autophagy
Source: J Cell Mol Med. 2023 Mar 15;27(7):939–49. doi: 10.1111/jcmm.17700 (PMC10064033; doi:10.1111/jcmm.17700)
Supplement: Supplementary file 1 — Appendix S1. [file JCMM-27-939-s001.docx]

**Supplemental figures**

**Figure 1.**

**
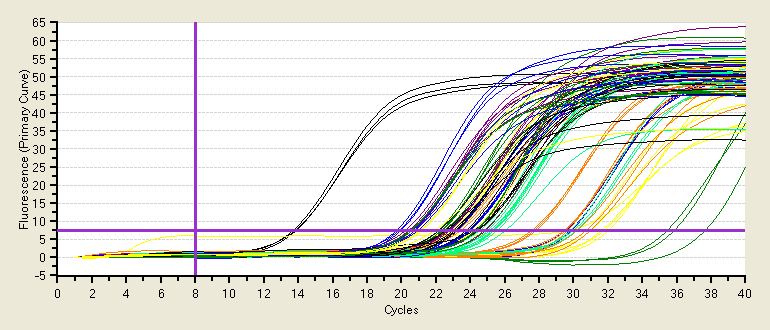
**

58 genes expressed in lung cancer PC-9 cells by RT-PCR analysis.

**Figure 2.**


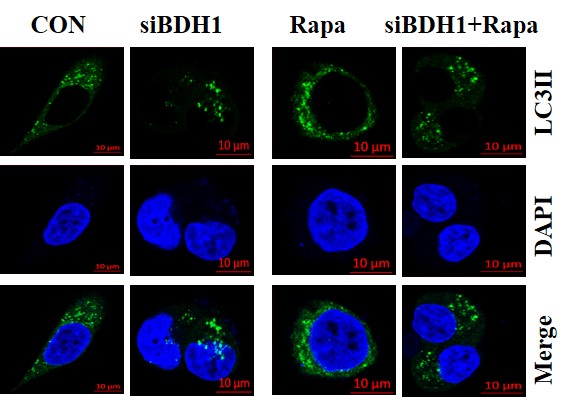


Immunofluorescence determined LC3-Ⅱ expression after siRNA-BDH1 transfection and/or rapamycin treatment in PC-9 cells.

**Figure 3.**

**
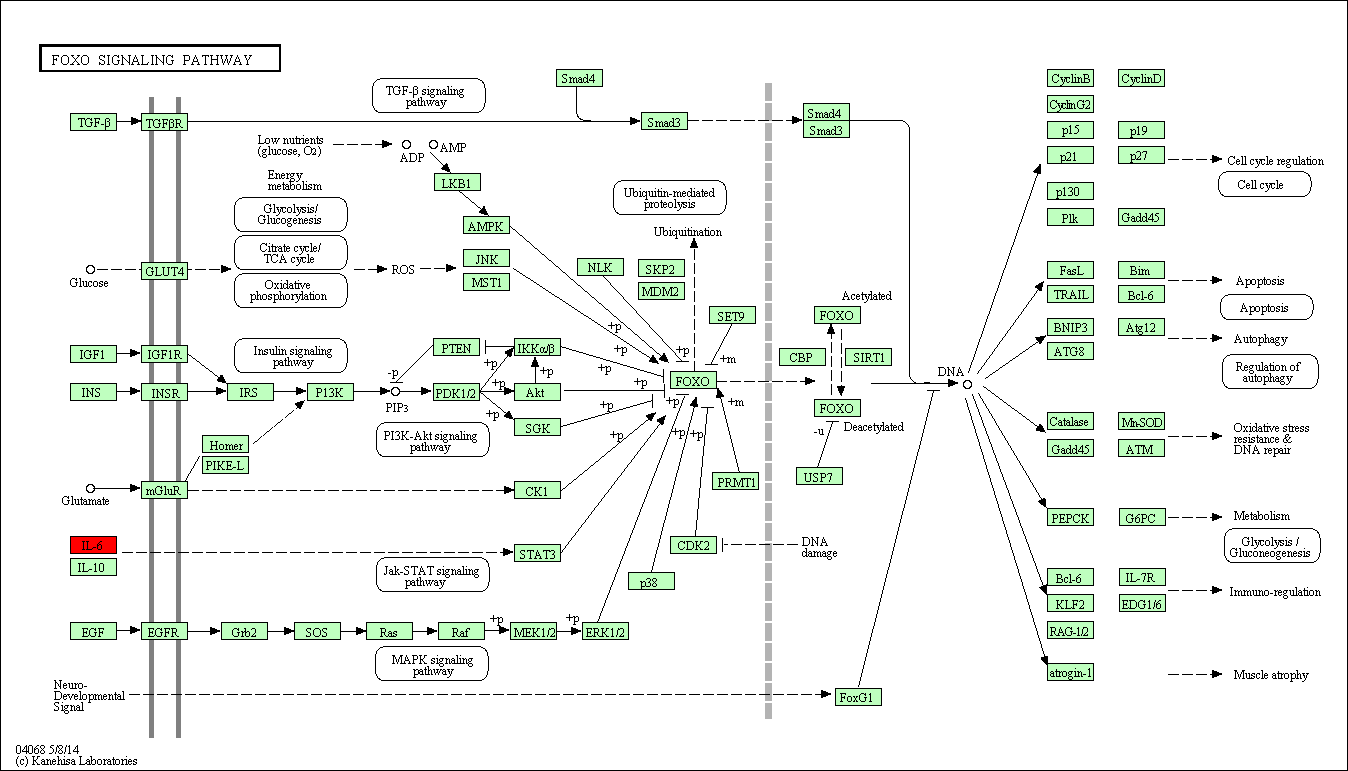
**

Data represent of singling pathway in KEGG.

**Figure 4.**

**
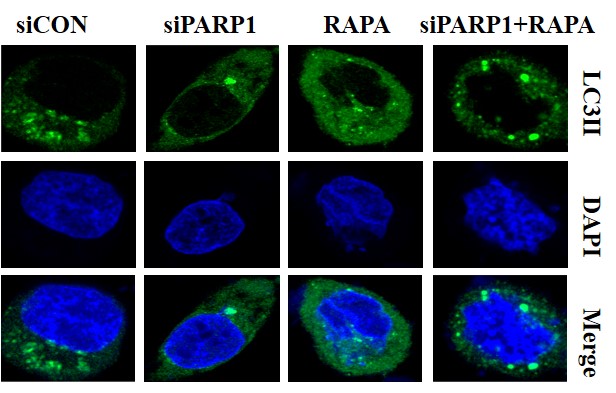
**

Immunofluorescence determined LC3-Ⅱ expression after siRNA-PARP1 transfection and/or rapamycin treatment in PC-9 cells.

**Supplemental tables**

**Table 1.** Clinical characteristics of 110 lung cancer patients

|  | | Count | Column N % |
| --- | --- | --- | --- |
| gender | Female | 62 | 33.3% |
|  | Male | 124 | 66.7% |
| Histology | Adenocarcinoma | 32 | 17.2% |
|  | Squmous and others | 154 | 82.8% |
| T | T1 | 42 | 22.6% |
|  | T2 | 119 | 64.0% |
|  | T3-T4 | 25 | 13.4% |
| N | N0 | 119 | 64.3% |
|  | N1-N3 | 66 | 35.7% |
| M | M0 | 176 | 96.7% |
|  | M1 | 6 | 3.3% |
| Stage | I-II | 143 | 77.7% |
|  | III-IV | 41 | 22.3% |

**Table 2.** Clinical characteristics of 66 lung cancer patients

|  | | Count | Column N % |
| --- | --- | --- | --- |
| Sex | Female | 20 | 30.3% |
|  | Male | 46 | 69.7% |
| T | 0 | 1 | 1.5% |
|  | 1 | 11 | 16.7% |
|  | 2 | 36 | 54.5% |
|  | 3 | 6 | 9.1% |
|  | 4 | 12 | 18.2% |
| N | 0 | 21 | 31.8% |
|  | 1 | 13 | 19.7% |
|  | 2 | 23 | 34.8% |
|  | 3 | 9 | 13.6% |
| M | 0 | 43 | 65.2% |
|  | 1 | 23 | 34.8% |

**Table 3.** KEGG pathway of DEGs with corrected P< 0.0001 and expression in more than half of the samples.

| **Term** | **Database** | **Sample number** | **Background number** | **P-Value** | **Corrected P-Value** |
| --- | --- | --- | --- | --- | --- |
| PI3K-Akt signaling pathway | KEGG PATHWAY | 8 | 347 | 3.45E-05 | 7.76E-04 |
| Metabolic pathways | KEGG PATHWAY | 7 | 1218 | 0.2036 | 2.91E-01 |
| Focal adhesion | KEGG PATHWAY | 7 | 207 | 9.26E-06 | 3.12E-04 |
| Complement and coagulation cascades | KEGG PATHWAY | 7 | 69 | 5.23E-09 | 7.06E-07 |
| Pathways in cancer | KEGG PATHWAY | 7 | 398 | 0.000529 | 5.49E-03 |
| Phagosome | KEGG PATHWAY | 5 | 155 | 0.000216 | 2.92E-03 |
| ECM-receptor interaction | KEGG PATHWAY | 5 | 87 | 1.39E-05 | 3.76E-04 |
| Cell adhesion molecules | KEGG PATHWAY | 5 | 145 | 0.000159 | 2.68E-03 |
| Tight junction | KEGG PATHWAY | 5 | 138 | 0.000126 | 2.43E-03 |
| Malaria | KEGG PATHWAY | 5 | 49 | 8.01E-07 | 5.41E-05 |

**Table 4.** Clinical characteristics of 188 lung cancer patients from the TCGA dataset

|  | | Count | Column N % |
| --- | --- | --- | --- |
| gender | Female | 58 | 33.3% |
|  | Male | 124 | 66.7% |
| Histology | Adenocarcinoma | 32 | 17.2% |
|  | Squmous and others | 154 | 82.8% |
| T | T1 | 42 | 22.6% |
|  | T2 | 119 | 64.0% |
|  | T3-T4 | 25 | 13.4% |
| N | N0 | 119 | 64.3% |
|  | N1-N3 | 66 | 35.7% |
| M | M0 | 176 | 96.7% |
|  | M1 | 6 | 3.3% |
| Stage | I-II | 143 | 77.7% |
|  | III-IV | 41 | 22.3% |

**Table 5**. Clinical characteristics of 10 lung cancer patients for the genome-wide exon sequencing of lymph node blocks

| patients | SEX | Age | ID | Smoking | Pathological | lymph node metastasis |
| --- | --- | --- | --- | --- | --- | --- |
| number |  |  |  |  |  |  |
| 1 | male | 58 | 744472 | NO | Adenocarcinoma | NO |
| 2 | male | 51 | 775032 | NO | Adenocarcinoma | NO |
| 3 | male | 70 | 775772 | NO | Adenocarcinoma | NO |
| 4 | male | 73 | 770449 | NO | Adenocarcinoma | NO |
| 5 | male | 58 | 761502 | NO | Adenocarcinoma | NO |
| 6 | male | 64 | 734080 | NO | Adenocarcinoma | YES |
| 7 | male | 68 | 740786 | NO | Adenocarcinoma | YES |
| 8 | male | 66 | 270216 | NO | Adenocarcinoma | YES |
| 9 | male | 67 | 764199 | NO | Adenocarcinoma | YES |
| 10 | male | 68 | 760026 | NO | Adenocarcinoma | YES |

**Table 6.** Nine genes, T-stage, were found to be associated with lymph node metastases

| Variables not in the Equation | | | |
| --- | --- | --- | --- |
| Variables | Score | df | Sig. |
| DDX49 | 7.75 | 1 | 0.01 |
| USH1G | 4.839 | 1 | 0.03 |
| MRM1 | 3.565 | 1 | 0.06 |
| RBM28 | 6.48 | 1 | 0.01 |
| USP49 | 4.879 | 1 | 0.03 |
| BDH1 | 6.087 | 1 | 0.01 |
| EGFR | 6.394 | 1 | 0.01 |
| ABHD11 | 7.448 | 1 | 0.01 |
| GTPBP3 | 10.274 | 1 | 0 |
| age | 1.884 | 1 | 0.17 |
| gender | 3.217 | 1 | 0.07 |
| Histology | 1.14 | 1 | 0.29 |
| T-Stage | 44.874 | 1 | 0 |
| Overall Statistics | 69.099 | 13 | 0 |
